# Supplementary Effects of *Allium hookeri* Extract on Glucose Tolerance in Prediabetic Subjects and C57BL/KsJ-*db/db* Mice

**DOI:** 10.3390/ph16101364

**Published:** 2023-09-27

**Authors:** Ji-Su Kim, Hyun-Ju Kim, Eun-Byeol Lee, Ji-Hye Choi, Jieun Jung, Hwan-Hee Jang, Shin-Young Park, Ki-Chan Ha, Yu-Kyung Park, Jong-Cheon Joo, Sung-Hyen Lee

**Affiliations:** 1 Functional Food Division, Department of Agro-food Resources, National Institute of Agricultural Sciences, Rural Development Administration, Wanju 55365, Jeonbuk, Republic of Korea; ver0218@korea.kr (J.-S.K.); dmsqufdl1029@naver.com (E.-B.L.); jyyye@jbnu.ac.kr (J.-H.C.); jjempm@korea.kr (J.J.); rapture19@korea.kr (H.-H.J.); soyoenj@korea.kr (S.-Y.P.); 2 Kimchi Functionality Research Group, World Institute of Kimchi, Gwangju 61755, Jeolla, Republic of Korea; hjkim@wikim.re.kr; 3 Healthcare Claims & Management Incorporation, Jeonju 54858, Jeonbuk, Republic of Korea; alswl5592@gmail.com (K.-C.H.); yukyungpark07@gmail.com (Y.-K.P.); 4 Department of Sasang Constitutional Medicine, College of Korean Medicine, Wonkwang University, Iksan 54596, Jeonbuk, Republic of Korea; jcjoo@wku.ac.kr

**Keywords:** *Allium hookeri*, prediabetes, blood glucose, insulin, area under the curve of glucose and insulin, glycemia, insulin resistance

## Abstract

*Allium hookeri* (AH) has been used as a nutritional and medicinal food in Asia for many years. Our previous studies have described its anti-diabetic, anti-obesity, and anti-inflammatory activities in animal models and prediabetes. This study investigated whether AH could improve glycemia by modulating insulin secretion in prediabetic subjects through an in-depth study. Eighty prediabetic subjects (100 ≤ fasting plasma glucose < 140 mg/dL) were randomly assigned to a placebo (*n* = 40) group or an ethanol AH extract (500 mg/day, *n* = 40) group for 12 weeks. Dietary intake and physical activity, blood glucose (an oral glucose tolerance test for 120 min), insulin (insulin response to oral glucose for 120 min), area under the curve (AUC) of glucose or insulin after oral glucose intake, insulin sensitivity markers, C-peptide, adiponectin, glycated hemoglobin A1c (HbA1c) levels, hematological tests (WBC, RBC, hemoglobin, hematocrit, and platelet count), blood biochemical parameters (ALP, AST, total bilirubin, total protein, albumin, gamma-GT, BUN, creatinine, LD, CK, and hs-CRP), and urine parameters (specific gravity and pH) were examined at both baseline and 12 weeks after supplementation with placebo or AH capsules. Fifty-eight participants (placebo group: 20 men and 10 women; AH group: 13 men and 15 women) completed the study. AH supplementation moderately reduced postprandial blood glucose at 60 min (−6.14 mg/dL, *p* = 0.061), postprandial insulin levels at 90 min (−16.69 µU/mL, *p* = 0.017), the glucose AUC at 90 min (−412.52 mg*min/dL, *p* = 0.021), as well as the insulin AUC at 90 min (−978.77 µU*min/mL, *p* = 0.021) and 120 min (−1426.41 µU*min/mL, *p* = 0.015) when compared with the placebo group. However, there were no effects of AH on dietary intake and physical activity; HOMA index; HbAlc; C-peptide; or adiponectin, hematological-, blood biochemical-, and urinary markers. To confirm the effects of AH extract on blood glucose insulin sensitivity, C57BL/6J or C57BL/KsJ-*db/db* mice were used (*n* = 8/group). Body weight, fasting plasma glucose level, lipid profiles, liver and renal function, pancreatic histology, and insulin immunoreactivity were assessed. In the diabetic *db/db* mice, hyperglycemia, which was accompanied by an increase in insulin secretion in diabetic mice, was significantly reduced by AH treatment, resulting in the alleviation of β-cell overcompensation and insulin resistance. We confirmed that AH supplementation can effectively control blood glucose and insulin levels by improving insulin sensitivity and may be a potential agent for glycemic control in subjects with prediabetes and type 2 diabetes mellitus.

## 1. Introduction

Type 2 diabetes mellitus (T2DM) is characterized by hyperglycemia and hyperinsulinemia due to excess hepatic glucose production and insulin resistance [1]. The incidence rate of diabetes is increasing globally, and it is assumed that the number of patients with T2DM will increase to 700 million by 2045 [2]. Diabetes care is cost-effective when timely diagnosis enables early treatment [3]. Therefore, dietary and lifestyle modifications are recommended as primary strategies for T2DM prevention and treatment [4]. Furthermore, the intake of nutritional phytochemicals is considered an effective and practical therapeutic strategy [5].

Augmented postprandial glucose (PPG) levels are a hallmark of diabetes, and lowering PPG levels is crucial in T2DM treatment [6]. Hypoglycemic agents, such as acarbose, miglitol, and voglibose, decrease PPG mainly by reducing the rate of carbohydrate digestion and gastrointestinal glucose uptake and improving hyperglycemia and insulin sensitivity in T2DM [7]. Insulin, the essential glucoregulatory hormone, modulates glucose uptake from the blood into the liver, fat, and skeletal muscle. Moreover, pancreatic β-cells have evolved to possess immense plasticity for secretory adaptation to nutrient status [8,9]. Insulin resistance is perceived as a pivotal factor in T2DM pathogenesis, promoting a compensatory response of pancreatic β-cell enlargement that results in hyperinsulinemia [10,11]. Therefore, several anti-diabetic drugs have been widely used to control high blood glucose levels in T2DM; however, these are related to adverse effects, such as hypoglycemia, vomiting, and hepatotoxicity. Hence, using agents from natural foods that can avoid such side effects while controlling glycemia is increasingly sought after [5]. Research on alternative therapies has evaluated many plant extracts highlighted as candidate formulations for diabetes management.

*Allium hookeri* Thwaites (Liliaceae family) (AH), a vegetable widely used as a medicinal food in Asia [12], possesses anti-diabetic [13,14], anti-obesity [15,16], antioxidant [17], anti-inflammatory [18,19], and neuroprotective [20,21] activities. The main active compounds of AH root are alliin and cycloalliin, which reportedly decrease blood glucose, hemoglobin A1c (HbA1c), leptin levels, and the oxidative stress-induced inflammatory response in diabetic animals and prediabetes [13,14,22,23]. AH ethanolic extract exhibited greater efficacy against hyperglycemia than a hot water extract of AH (AHW) in diabetic mice [22]. However, most AH-probing studies for anti-diabetic effects have been conducted in animal models. Our previous trial evaluated the hypoglycemic effect of AHW (243 mg/d) supplementation for 8 weeks in subjects with prediabetes and was the only study conducted with a crossover clinical design. Therefore, through in-depth research, we explored the effects of AH extracts on glycemia, insulin levels, and insulin resistance in animal models and prediabetic subjects supplemented with AH for 12 weeks.

## 2. Results

### 2.1. Concentration of Cycloalliin

**Figure 1:**
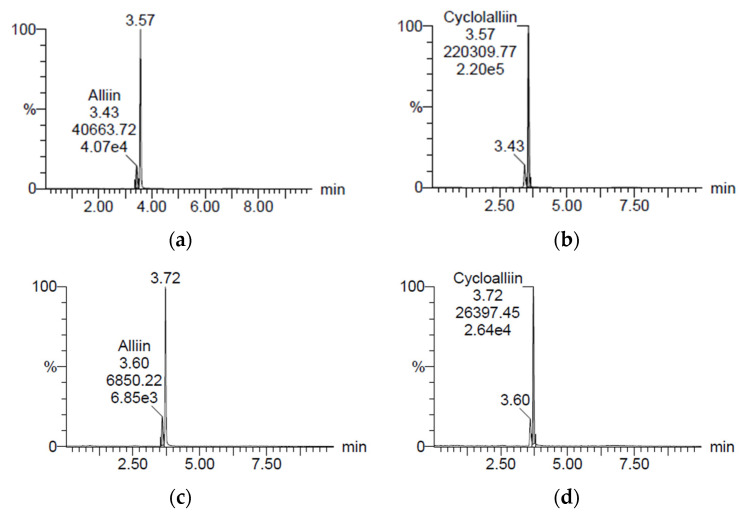
Chromatograms of (**a**) alliin and (**b**) cycloalliin in ethanol extract and (**c**) alliin and (**d**) cycloalliin in hot water extract of *A. hookeri*, analyzed by LC/MS.

Figure 1 reveals the concentrations of alliin and cycloalliin (C_6_H_11_NO_3_S) in the ethanol (AH) and hot water (AHW) extracts of *A. hookeri*. They were analyzed by a research team from Jeonbuk National University (Jeonju) and the Korea Functional Food Institute (Sung Nam, Republic of Korea). The alliin and cycloalliin concentrations were 1.39 and 3.17 mg/g in AH (Figure 1a,b), while the levels were 0.62 and 2.06 mg/g in AHW (Figure 1c,d), respectively. Alliin and cycloalliin concentrations were higher in AH than in AHW.

### 2.2. Clinical Study

#### 2.2.1. Clinical Demographic Characteristics of the Trial Subjects

**Figure 2:**
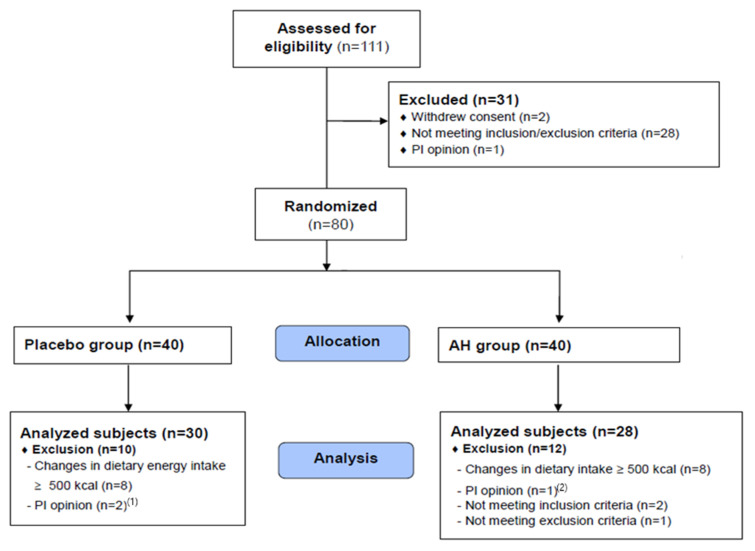
CONSORT diagram showing the study flow. ^(1)^ Plasma triglyceride concentration > 500 mg/dL, ^(2)^ HbA1c concentration > 8%.

A total of 111 volunteers (potential study subjects) signed a written informed consent form and then participated in a screening test to select 80 people who met the eligibility criteria. Finally, 30 and 28 subjects in the placebo and AH groups, respectively (>72.5%), completed the study (Figure 2).

General characteristics, including sex, age, height, weight, body mass index (BMI), systolic blood pressure (SBP), diastolic blood pressure (DBP), pulse, alcohol consumption, smoking status, and family history of diabetes, are listed in Table 1 and were not significant between the placebo and AH groups. In this study, 33 subjects were male (20 subjects in the placebo group and 13 subjects in the AH group), and 25 subjects were female (10 subjects in the placebo group and 15 subjects in the AH group). The average age was 52.1 ± 8.1 years and 50.3 ± 8.2 years in the placebo and AH groups, respectively. The mean height was 166.1 ± 8.5 cm and 165.7 ± 8.3 cm in the placebo and AH groups, respectively. The average body weight was 70.5 ± 12.1 kg and 72.2 ± 12.7 kg, and the mean BMI was 25.4 ± 2.9 kg/m^2^ and 26.1 ± 2.8 kg/m^2^ in the placebo and AH groups, respectively. The mean SBP was 125.4 ± 14.7 and 122.8 ± 9.8 mmHg, the mean DBP was 78.2 ± 10.3 and 76.3 ± 8.2 mmHg, and the average pulse rate was 70.3 ± 6.7 beats/min and 70.3 ± 8.6 beats/min in the placebo and AH groups, respectively. There were 31 alcohol drinkers (19 in the placebo group and 12 in the AH group) and 15 smokers (9 in the placebo group and 6 in the AH group). Subjects with a family history of diabetes accounted for 30% and 50% in the placebo and AH groups, respectively, without a significant difference (*p* > 0.05). All safety-related parameters, including electrocardiogram, vital signs, and laboratory test results, were within the normal range throughout the experimental period. There was no significance between the two experimental groups.

#### 2.2.2. Nutrient Intake and Physical Activity 

Nutrient intake and physical activity are shown in Table 2. There were no significant differences in carbohydrate, fat, protein, or dietary fiber intake between the placebo and AH groups. SBP, DBP, and pulse values were similar in the placebo and AH groups before and after 12 weeks of AH treatment. However, unexpected physical activity which was measured by metabolic equivalent of task (T-MET) was lower by 15 and 43.1% in the AH group than in the placebo group at baseline and after 12 weeks of treatment, respectively. There was no significant difference (*p* > 0.05).

#### 2.2.3. Comparison of Plasma Glucose, Insulin Levels, and Related Factors 

The effects of AH supplementation on plasma glucose and insulin levels under fasting conditions during the oral glucose tolerance test (OGTT) and other metabolic parameters are shown in Table 3. Generally, plasma glucose and insulin levels at 30, 60, and 90 min after oral glucose intake were lower in the AH group compared with the placebo group. Their decreases from baseline to 12 weeks after supplementation with AH were higher in the AH group. A decrease of >3.1% in PPG was observed in the AH group at 60 min during the OGTT (*p* = 0.061), with changes of 8.4 mg/dL in the placebo group and −6.14 mg/dL in the AH group.

**Figure 3:**
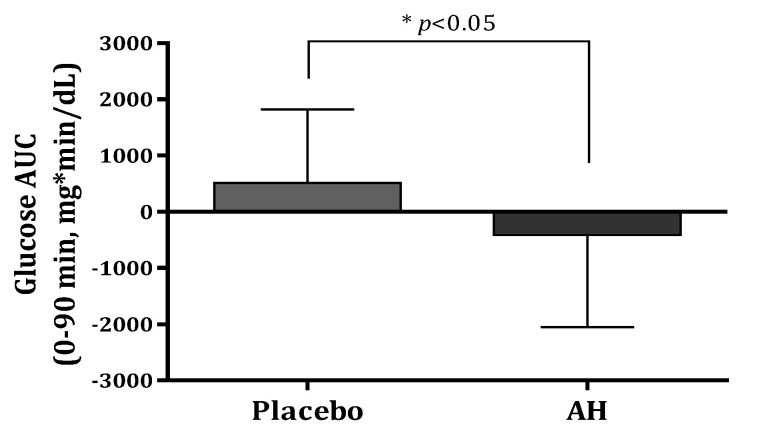
Comparison of glucose AUC (0–90 min) levels after oral glucose treatment between placebo and AH groups. AUC, area under the curve. Data are presented as the mean ± SD. * AH group is significantly different from the placebo group at *p* < 0.05 by *t*-test.

AH treatment decreased the area under the curve (AUC) of glucose at 0–30 min, 0–60 min, 0–90 min, and 0–120 min during the OGTT by 93.21, 285.54, 412.52, and 395.91 mg*min/dL, respectively. Compared with the values at baseline, the glucose AUCs for 120 min after glucose intake exhibited 4.6–8% reductions in the AH group. The glucose AUC significantly decreased at 0–90 min compared with that of the placebo group (*p* = 0.021) at Figure 3. The glucose Cmax decreased by 4.46 mg/dL in the AH group (*p* > 0.05) after the AH supplement for 12 weeks.

**Figure 4:**
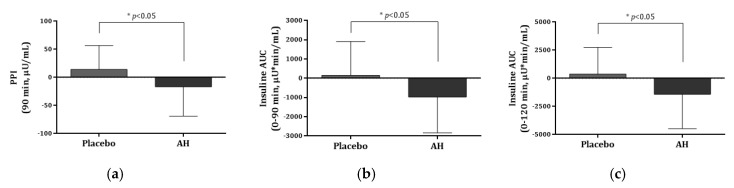
Comparison of insulin and AUC levels between the placebo and AH groups. (**a**) PPI at 90 min, (**b**) insulin AUC (0–90 min), and (**c**) insulin AUC (0–120 min) levels after oral glucose treatment. PPI, postprandial plasma insulin; AUC, area under the curve. Data are presented as the mean ± SD. * AH group is significantly different from the placebo group at *p* < 0.05 by *t*-test.

AH supplementation decreased the insulin AUC at 0–30 min, 0–60 min, 0–90 min, and 0–120 min by 236.57, 632.09, 978.77, and 1426.41 during the OGTT. Compared with the baseline, the AH group’s values were significantly lower (*p* > 0.02). Between the two groups, there were significant differences found in the insulin AUC (0–90 min) and AUC (0–120 min) at 90 min and 120 min after oral glucose treatment, with the values being significantly lower in the AH group compared with those in the placebo group (*p* = 0.021, 0.015). AH supplementation also significantly reduced the insulin Cmax by 14.21 μU/mL (12.2%) compared with baseline in the AH group, indicating a significant decrease in the plasma insulin concentration during the OGTT in the AH group (*p* = 0.033). However, there were no significant differences in HOMA-IR, HOMA-β, QUICKI, AIR, HbA1c, C-peptide, or adiponectin between the placebo and AH groups. Plasma insulin levels during the OGTT were lower by 10.3–21.2% in the AH group after 12 weeks of AH supplementation compared with baseline. A significant difference was detected between the placebo and AH groups at 90 min after glucose intake (*p* = 0.017) at Figure 4. 

The hematological and blood biochemical parameters and urine data are shown in Table 4. After 12 weeks of placebo and AH supplementation, no significant difference in hematological parameters was observed between the two groups. Indicators of liver function (ALP, AST, ALT, total bilirubin, total protein, albumin, gamma-GT), kidney function parameter (creatinine), tumor marker (LD), markers of cardiac injury (CK, cardiovascular disease risk factor (hs-CRP), as well as urine gravity and pH were similar between the placebo and AH groups after AH supplementation. BUN levels increased from 13.48 to 14.69 in the placebo group and decreased from 13.74 to 12.57 in the AH group. However, the BUN values in 12 weeks were not significantly changed from the values at the baseline in both groups (*p* > 0.07).

### 2.3. Animal Study

**Figure 5:**
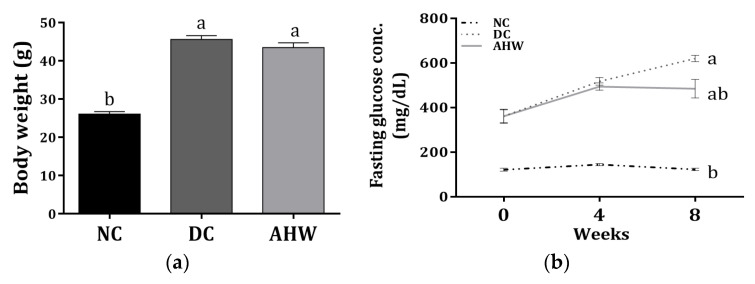
Effects of AHW treatment for 8 weeks on insulin sensitivity in C57BL/KsJ-*db/db* mice. (**a**) Body weight and (**b**) changes of fasting plasma glucose concentrations. Data are presented as the mean ± SEM (*n* = 8). ^a,b^ Different letters are significantly different at *p* < 0.05. NC, normal control; DC, diabetic control; AHW, diabetic mice fed water extract of AH.

Figure 5 shows the body weight and fasting plasma glucose (FPG) levels in the experimental normal and diabetic mice treated with or without AHW for 8 weeks. The diabetic control DC group administered with distilled water (D.W.) presented a marked increase in body weight compared with the normal control group (NC), while the body weight decreased with AHW treatment (Figure 3a). As expected, the FPG levels significantly increased in the DC group compared with the NC group after 8 weeks of experiment; however, the levels decreased in the AHW group at 8th week but not the 4th week (Figure 3b).

The concentrations of FPG, lipid concentrations and hepatic and kidney functional parameters were shown in Table 5. Plasma triglyceride (TG), total-cholesterol (TC), and LDL-cholesterol (LDL-C) levels, which increased in the diabetic mice, were significantly lower in the AHW group than in the DC group without changes in HDL-cholesterol (HDL-C) level. The hepatic and kidney functional parameters, including plasma AST, ALT, urea nitrogen, and Cr levels were higher in the DC group than the NC group. There was no significant difference between the DC and AHW groups.

**Figure 6:**
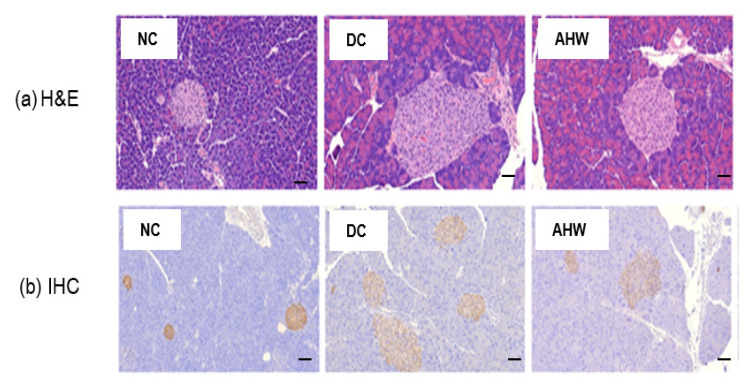
Effects of AHW treatment for 8 weeks on insulin sensitivity in C57BL/KsJ-*db/db* mice. (**a**) representative pancreatic H&E (upper panel) and (**b**) IHC (insulin, lower panel) staining. Scale bar = 100 um. NC, normal control; DC, diabetic control; AHW, diabetic mice fed water extract of AH.

Figure 6a shows the marked increases in pancreatic islet number and size in the DC group compared with the NC group, as shown by H&E staining. The AHW group showed a significant improvement in these abnormal histological phenomena. The IHC results indicated that the insulin response was higher in the DC group than the NC group (Figure 6b). The AHW group showed less immunoreactivity to insulin than the DC group, indicating that AH can prevent hyperglycemia induced by blunting β-cell overcompensation and insulin resistance in type 2 diabetic mice.

## 3. Discussion

Prediabetes defined as hyperglycemia is below the pathologic threshold [HbA1c 5.7–6.4%, and/or FPG 100 mg/dL to 125 mg/dL] but almost always precedes type 2 diabetes [24]. In many randomized controlled clinical trials, lifestyle interventions are effective in preventing the progression of prediabetes to diabetes [14,25]. Clinical trials to evaluate hypoglycemic effects of functional foods have been allowed only in prediabetes, but not in diabetic patients in Korea. In this study, AH supplementation significantly decreased glucose AUC (0–90 min) in Figure 3, PPI at 90 min, and insulin AUCs at 0–90 min and 0–120 min in Figure 4 in the subjects with prediabetes and AH administration alleviated the increase of plasma glucose and lipid levels and pancreatic insulin secretion in *db/db* mice. The results indicate that AH may prevent hyperglycemia by alleviating β-cell overcompensation and insulin resistance.

AH has various beneficial properties, such as anti-diabetic [13,14,22,23], anti-inflammatory [18,19], and anti-obesity [15,16,26] effects. In addition, AH contains diverse phenols, phytosterols, and high amounts of sulfur compounds, including methiin and cycloalliin, which are stable and odorless cyclic compounds formed by alliin during the cooking process [27]. Cycloalliin has been extensively studied as a functional compound [17]. Many studies have reported that cycloalliin from Allium plants exhibits antioxidant [17,28], hypolipidemic [29,30], anti-obesity [31], and fibrinolytic [32] activities. Studies with a pure cycloalliin in the amount identical to what was in the encapsulated extract in future trial can be helpful to explain the material’s anti-diabetic effect.

In our previous study, the hypoglycemic effects of AH were accompanied by a decrease in blood glucose levels at 60 min after oral glucose treatment and significant reductions in the AUC and HbA1c of subjects with prediabetes following an 8-week crossover trial [14]. However, in this study, there was no significance found in FPG and HbAlc after 12 weeks of placebo and AH treatment. This might be a result, at least in part, of lower physical activity in the AH-supplemented group, although we did not observe such a significant difference. Despite having no effects on HbA1c, the inhibitory effect of AH on postprandial glycemia, as indicated by a decrease in glucose AUC and PPI at 90 min, supports the proposed beneficial effects of AH on insulin resistance in subjects with prediabetes. T2DM is characterized by impaired glucose homeostasis and a β-cell compensation response, which results in increased insulin secretion due to insulin resistance in the pancreas, ultimately culminating in β-cell death [33,34]. Insulin resistance is referred to as decreased insulin sensitivity, which compromises insulin-induced glucose uptake in muscle and adipose tissue and hepatic gluconeogenesis [35]. Compounds with antioxidant properties protect against overcompensation and loss of pancreatic β-cells during T2DM progression [36]. On the other hand, Imeglimin, an anti-diabetic drug, improved insulin secretion and pancreatic β-cell function by increasing the number of insulin granules and reducing apoptotic β-cell death in T2DM [37,38]. Taken together, AH has been widely consumed as a food in Eastern Asia for over 500 years; therefore, it is safe and may be useful as a supplement for controlling blood insulin levels by modulating insulin secretion in pancreatic β-cell in prediabetic subjects.

PPG values during the OGTT strongly predicted HbA1c in patients with and without diabetes [39]. Furthermore, glycemic control drugs lowered PPG and PPI by reduction of carbohydrate digestion and gastrointestinal glucose uptake [7,40]. Previously suggested mechanisms for the anti-diabetic effect of AH postulate that it protects pancreatic β-cells from oxidative damage [13], and improves insulin sensitivity [22]. As another mode of action, glucose-6-phosphatase, sterol regulatory element-binding protein-1, acetyl CoA carboxylase, and fatty acid synthase protein levels decreased in the liver of *db/db* diabetic mice, suggesting an inhibitory effect on gluconeogenesis and fatty acid synthesis in the AHW group (Figure S1). It was partially explained by the modulated carbohydrate metabolism due to enriched functions of microorganisms [41] and by the inhibitory effects on gluconeogenesis and fatty acid synthesis due to controlling IRS-1, GLUT4 and fatty acid synthase protein levels [23,42,43]. In our previous 8-week crossover clinical trial, the AH supplementation group exhibited significantly lower plasma glucose at 60 min of the OGTT and AUC and HbA1c levels than those in the placebo group, suggesting that AH might be effective for chronic glycemic control [14]. Table 4 shows that insulin Cmax, Tmax, and C-peptide levels decreased in the AH group, suggesting that the glucose-lowering effects of AH were not due to the promotion of insulin secretion but to reduced insulin resistance. In our previous study, AH extracts alleviated hyperglycemia through controlling FPG, glucose tolerance, and insulin sensitivity in T2DM mice. The effects were comparable with those of metformin, which is widely used as a drug for diabetes [22]. AH leaves also prevented impaired glucose metabolism and stimulated glutathione biosynthesis in T2DM rats, attributed to the organosulfur compound alliin [23]. FPG and insulin levels decreased significantly with AH in T2DM mice, indicating that AH improves insulin sensitivity [22]. HOMA has been analyzed to evaluate β-cell function and insulin resistance (IR) from FBG and insulin concentrations. In this study, there was no significant difference was found in HOMA levels between AH and placebo groups (Table 5). It may be explained that HOMA index varies according to the demographic characteristics of the subjects and the data needs to be interpreted carefully when the difference in primary baseline data of the subjects [44,45]. In other study, AH significantly decreased the serum glucose level in type I diabetic mice, potentially through insulin release from the β-cells of islets by preventing oxidative damage and inflammation [13]. These findings suggest that AH may benefit metabolic abnormality by controlling PPG and PPI levels by reducing diabetes risk factors.

Modifying PPG with foods or drugs is well-known to reduce the risk of T2DM [39]. Mulberry leaf promoted PPG control in subjects with T2DM, and the observed reduction in the PPG response was implicated in decreased insulin secretion [46]. These results support our current findings that sulfur-containing compounds from garlic exert direct hypoglycemic effects by reducing fasting blood glucose, PPG, and plasma insulin levels while increasing hepatic glycogen synthesis in diabetic animal models and patients with T2DM [47,48,49]. In addition, a meta-analysis reported that garlic supplementation positively affected blood glucose, glycated hemoglobin, and lipid levels to manage T2DM [50]. Our results confirmed previous data on AH supplementation in prediabetic subjects in an 8-week crossover trial [14]. Thus, AH may be a potential agent for glycemic control in prediabetic patients.

A weak point of this study is that physical activity expressed as T-MET was not controlled for 12 weeks but, significance was not reached between the two groups. The T-MET in the placebo group increased from 1883.3 to 2080 min/week compared with 1600 to 1183.6 min/week in the AH group. The changes in T-MET levels between 0 weeks (baseline) and 12 weeks in placebo and AH groups were +196.7 and −416.4 min/week, indicating an increase and decrease of 10.5% and 26.0% in the groups, respectively. And an in-depth phytochemical analysis in future studies would allow us to better understand the anti-glycemic effects of AH. Despite these limitations, the AH supplementation evaluated in this study improved plasma PPG and PPI levels without any side effects or hypoglycemia in subjects with prediabetes. Further studies should be carried out to clarify the mechanisms in diabetic subjects treated with AH supplements.

## 4. Materials and Methods

### 4.1. Samples for Human and Animal Trials

For the clinical study, the product (AH) was extracted from AH roots using 10 times the volume of 50% *v*/*v* ethanol at 40 °C for 8 h, concentrated at 65 °C and 650 mH (HS Tech., Daegu, Republic of Korea), and freeze-dried (PVTFD 300R, Ilsin Lab., Yangju, Republic of Korea). Each AH capsule contained 500 mg of AH and diluting ingredients. By contrast, the capsule for the placebo group contained microcrystalline cellulose, silicon dioxide, caramel color, and red color and had the same volume as the AH capsule (Table 6).

For the animal study, dried AH roots were purchased from the Hanam company in Sunchang, Jeonbuk Province (Republic of Korea). AHW was extracted from the AH using 10 times the volume of water at 95 °C for 12 h, filtered with paper No. 2 (Whatman International, Kent, UK), freeze-dried (PVTFD 10R, Ilsin Lab.), and pulverized.

Alliin and cycloalliin concentrations were measured by the method reported in the previous study [21]. Two extracts, alliin (L-Alliin, Sigma-Aldrich Co., St. Louis, MO, USA) as a standard and cycloalliin hydrochloride monohydrate (Fujifilm Wako Pure Chemical Co., Osaka, Japan) as a standard, were dissolved in methanol at 0.1 mg/mL. LC/MS (Agilent 6410 Triple Quad, Agilent Technologies, Santa Clara, CA, USA) coupled to an MS QQQ mass spectrometer with electrospray ionization (ESI) was used. Quantifications were performed using multiple reaction monitoring (MRM) of the transitions of *m*/*z* 178 → 88 for alliin and *m/z* 178 → 73 for cycloalliin. A reversed phase C18 with polar end-capping (2 mm × 150 mm, Synergi^TM^ 4 μm Hydro-RP 80 Å; phenomenex, Torrance, CA, USA) was used for chromatographic separations. Solutions of 0.1% formic acid in water and 0.1% formic acid in acetonitrile were prepared as mobile phase A and B. The gradient system was set as 5% B at 0 min; 5% B at 1 min; 100% B at 11 min; 100% B at 12 min; 5% B at 15 min; and 5% B at 20 min. The operating temperature was 30 °C, and the flow rate was 0.2 mL/min. The gas temperature, gas flow, nebulizer, and capillary were 300 °C, 11 L/min, 15 psi, and 4000 V, respectively.

### 4.2. Clinical Study

This study was conducted as a randomized, double-blind, placebo-controlled trial over 12 weeks. Random assignment was blinded for all participants and staff. The protocol for this clinical trial was approved by the Institutional Review Board of WonKwang University Oriental Medicinal Hospital, Jeonju (IRB No.: WUJKMH-IRB-2021-0007). This project was registered at the clinical registration site (CRIS) at the start of the clinical trial, and the CRIS registration number was KCT0006365 (documented date: 22 July 2021). This study complied with the reporting trials standards (CONSORT) guidelines, the Declaration of Helsinki, and the International Conference on Harmonization (ICH-GCP). We modified the sample size by the changes in the test group’s FPG biomarker. A power calculation based on similar outcome variables and the design of a previous study [51] was applied. The predicted values in FPG after 12 weeks were 2.22 mg/dL in the placebo group and −6.28 mg/dL in the AH group, with a standard deviation of 11.24 mg/dL, statistical power of 80%, and α = 0.05. On the basis of the above assumptions, the total sample size was 80 (40 for each group) with a dropout rate of 30%. Before the trial, the entire process was expounded to all subjects, and consent was obtained. The subjects were randomly assigned to AH (*n* = 40) or placebo (*n* = 40) using SAS version 9.4 (SAS Institute, Cary, NC, USA) (Figure 1). The allocation number was concealed from the researchers. The participants were enrolled using sequentially numbered sealed envelopes. None of the subjects, researchers, investigators, or pharmacists knew the randomization until the study was terminated. The study’s sponsor maintained the safety and confidentiality of the master randomization list. During the trial period (14 July to 31 December 2021), participants were required to maintain their usual diet and activity. Subjects with changes in energy intake > 500 kcal, TG ≥ 500 mg/dL, and abnormal HbA1c (≥8%), which were considered factors that would influence the study (drinking and dietary intake before the visit), were excluded from the analysis on the basis of the PI’s opinion. The safety of AH intake was reviewed by analyzing hematological factors after supplementation for 12 weeks. Its anti-diabetic efficacy was assessed by comparing differences before and after taking the capsules for the same period and between the placebo and AH groups.

#### 4.2.1. Subjects

Subjects deemed as prediabetic (100 ≤ FPG < 140 mg/dL) according to the guidelines of the Ministry of Food and Drug Safety and who had not been diagnosed with any diseases were recruited from the WonKwang University Oriental Medicinal Hospital in Jeonju, and the trial was carried out as a double-blind study by the hospital.

The exclusion criteria at the time of screening for the study were as follows: (1) HbA1c ≥ 6.5%; (2) BMI < 18.5 kg/m^2^ or > 35 kg/m^2^; (3) clinically significant acute or chronic conditions, including cardiovascular, endocrine, hepatobiliary, renal and urinary, inflammatory, and gastrointestinal problems that require treatment; (4) blood sugar-related medicines, such as blood sugar-lowering, anti-obesity, and lipid-lowering drugs, up to 3 months before screening (refer to Prohibited Use of Drugs); (5) significant hypersensitivity reactions to ingredients; (6) anti-psychotic drug intake in the past 3 months; (7) confirmed or suspected alcoholism or substance abuse; (8) participation in another clinical trial in the past 3 months; (9) more than 3 times the reference ranges of serum AST and ALT as well as serum creatinine > 2.0 mg/dL; (10) pregnant or lactating women; and (11) subjects judged as unfit to participate in the study on the basis of the diagnostic laboratory test results or other reasons by the principal investigator.

#### 4.2.2. Evaluation of Diet and Physical Activity

Dietary intake and physical activity were evaluated during the 12-week study period to confirm whether there were any changes in the lifestyles of the participants. Dietary intake over 3 days was determined through the dietary record method using the CANpro 4.0 program (The Korean Nutrition Society, Seoul, Republic of Korea). Physical activity was calculated using a Global Physical Activity Questionnaire and expressed as T-MET. 

#### 4.2.3. Biochemical Measurements

At the end of the supplementation period, an electrocardiogram, vital signs (blood pressure and pulse rate), and laboratory tests (WBC, RBC, Hb, Hct, platelet count, ALP, AST, ALT, total bilirubin, total protein, albumin, gamma-GT, BUN, creatinine, LD, CK, and hs-CRP) were examined in the subjects for a safety evaluation. Hematological factors were assessed using the hematology analyzer KX-21N (Sysmex, Kobe, Japan), and other factors were determined by a 7180 Clinical Analyzer (Hitachi, Tokyo, Japan).

Blood glucose tests and OGTTs were performed before and after supplementation after overnight fasting. For the OGTT, blood samples were collected every 30 min, from 0 min after the intake of 75 g glucose, for 120 min. Blood glucose levels were evaluated using a 7180 Clinical Analyzer (Hitachi). The increase in AUC during the OGTT was assessed using the trapezoidal method [52]. Insulin and C-peptide concentrations were analyzed using a Cobas8000 e801 (Roche Diagnostics, Rotkreuz, Switzerland), and adiponectin concentrations were measured by a Spectramax190 (Molecular Devices, San Jose, CA, USA). The HbA1c concentration was analyzed using Cobas c513 (Roche Diagnostics, Rotkreuz, Switzerland).

### 4.3. Animal Study

Animal experiments were authorized by the Animal Research Committee of the World Institute of Kimchi (WIKIM-IACUC Approval No. 201501, 30 June 2015) and conducted according to the guidelines for the care and welfare of animals. Mice were obtained from Central Lab Animal Inc. (Seoul, Republic of Korea) and maintained at 22–24 °C and a relative humidity of 50 ± 10% with a 12 h light/dark lighting cycle. After a week of adaptation, the mice were divided into three groups (*n* = 8/group). C57BL/6J mice were orally administered with D.W. as NC group, and *db/db* mice were administered with D.W. (DC) or AHW at 100 mg/kg BW for 8 weeks. Body weight and food intake were recorded once a week. During the experiment, fasting blood samples were obtained for blood glucose measurement from the retro-orbital sinus by heparin-coated capillary tubes. At 8 weeks, the mice were fasted overnight and euthanized via CO_2_. Plasma concentrations of glucose, TG, TC, LDL-C, and HDL-C were evaluated using colorimetric enzymatic kits (Asan Pharm. Co., Ltd., Hwaseong, Republic of Korea). Plasma AST, ALT, urea nitrogen, and Cr levels were determined using commercial kits from Sigma-Aldrich (St. Louis, MO, USA). Pancreatic tissue was fixed and embedded in paraffin blocks, cut into 4-µm-thick sections, stained with hematoxylin and eosin (H&E), and histologically analyzed using a microscope (SV40; Olympus, Tokyo, Japan) at 100× magnification. For immunohistochemical analysis, the paraffin sections were deparaffinized, blocked with 5% skim milk in PBS, incubated with a mouse primary anti-insulin antibody, and stained with an ABC Kit (Vector Laboratories, Burlingame, CA, USA). Insulin reactivity was detected using a 3,3′-DAB Kit (Vector Laboratories), and the negative controls were incubated with 10% non-immune mouse serum.

### 4.4. Statistical Analysis

Data from animal experiments were analyzed using one-way analysis of variance followed by Duncan’s multiple range test (SPSS ver. 24, IBM Co., Armonk, NY, USA). All data from the human trials were analyzed using SAS version 9.4 (SAS Institute, Charlotte, NC, USA). The subject numbers were 30 and 28 for the placebo and AH groups, respectively, with a dropout rate of 30%. This study used a per-protocol approach and expressed the data as the means ± SD. Variables were analyzed using the chi-square test, and subsequent analysis of continuous variables used the *t*-test to determine the significance of differences between the groups. Statistical significance was set at *p* < 0.05. Figure 3, Figure 4 and Figure 5 were created using GraphPad Prism 6.01 (San Diego, CA, USA).

## 5. Conclusions

Our current findings highlight the protective effects of AH against β-cell overcompensation and insulin resistance in prediabetic subjects and T2DM mice. The observed reduction in glucose AUC, PPI, and insulin AUC levels may explain the alleviation of insulin resistance. The results suggest the efficacy of AH supplementation as a strategy for preventing hyperglycemia and other conditions associated with insulin resistance, including diabetes.

## Figures and Tables

**Table 1:** Demographic characteristics of the trial subjects.

	Placebo (*n* = 30)	AH (*n* = 28)	Total (*n* = 58)	*p*-Value ^(1)^
Sex (Male/Female)	20/10	13/15	33/25	0.120
Age (years)	52.1 ± 8.1	50.3 ± 8.2	51.2 ± 8.1	0.393
Height (cm)	166.1 ± 8.5	165.7 ± 8.3	165.9 ± 8.3	0.874
Weight (kg)	70.5 ± 12.1	72.2 ± 12.7	71.3 ± 12.3	0.615
BMI (kg/m^2^)	25.42 ± 2.9	26.1 ± 2.8	25.8 ± 2.8	0.364
SBP (mmHg)	125.4 ± 14.7	122.8 ± 9.8	124.1 ± 12.6	0.433
DBP (mmHg)	78.2 ± 10.3	76.3 ± 8.2	77.2 ± 9.4	0.440
Pulse (beats/m)	70.3 ± 6.7	70.3 ± 8.6	70.3 ± 7.6	0.979
Alcohol (n, %)	19 (63.3)	12 (42.9)	31 (53.5)	0.118
Smoking (n, %)	9 (30.0)	6 (21.4)	15 (25.9)	0.456
Family history of diabetes (n, %)	9 (30.0)	14 (50.0)	23 (39.7)	0.120

Values are presented as the mean ± SD or number (%). ^(1)^ Analyzed by chi-square test between the groups. BMI, body mass index; SBP, systolic blood pressure; DBP, diastolic blood pressure.

**Table 2:** Dietary intake and physical activity measured before and after supplementation with placebo and AH.

	Placebo (*n* = 30)				AH (*n* = 28)				*p*-Value ^(2)^
	Baseline	12 Weeks	Change Value	*p*-Value ^(1)^	Baseline	12 Weeks	Change Value	*p*-Value ^(1)^	
Energy (kcal)	1520.9 ± 491.2	1563.8 ± 449.6	42.9 ± 234.5	0.324	1507.4 ± 396.1	1485.9 ± 406.7	−21.5 ± 242.5	0.643	0.308
Carbohydrate (g)	199.5 ± 57.3	211.6 ± 46.1	12.2 ± 35.8	0.072	211.5 ± 51.9	217.0 ± 46.21	5.5 ± 41.3	0.488	0.511
Fat (g)	45.7 ± 21.6	44.0 ± 21.1	−1.7 ± 13.3	0.485	43.0 ± 14.1	38.1 ± 15.6	−4.9 ± 15.4	0.104	0.403
Protein (g)	68.0 ± 26.1	69.8 ± 24.8	1.8 ± 17.2	0.578	64.8 ± 21.0	62.0 ± 22.0	−2.8 ± 17.6	0.412	0.325
Dietary fiber (g)	17.1 ± 5.6	17.6 ± 5.1	0.5 ± 3.3	0.394	19.5 ± 6.2	19.4 ± 5.9	−0.1 ± 6.0	0.951	0.648
T-MET (min/week)	1883.3 ± 1691.4	2080.0 ± 3143.1	196.7 ± 3314.5	0.748	1600.0 ± 2231.3	1183.6 ± 1264.4	−416.4 ± 1411.4	0.130	0.360
SBP (mmHg)	126.43 ± 11.87	129.87 ± 11.56	3.43 ± 10.27	0.078	121.04 ± 12.99	127.39 ± 9.74	6.36 ± 14.27	0.026	0.372
DBP (mmHg)	76.03 ± 8.89	78.87 ± 9.58	2.83 ± 9.08	0.098	74.43 ± 11.43	78.39 ± 8.12	3.96 ± 10.09	0.047	0.655
Pulse (beats/m)	71.43 ± 5.57	70.33 ± 6.46	−1.10 ± 7.74	0.443	69.07 ± 8.89	71.00 ± 8.11	1.93 ± 8.52	0.242	0.162

Values are presented as the mean ± SD. ^(1)^ Analyzed by paired *t*-test between baseline and 12 weeks within each group. ^(2)^ Analyzed by independent *t*-test for changed values between the groups. T-MET, metabolic equivalent of task; SBP, systolic blood pressure; DBP, diastolic blood pressure.

**Table 3:** Plasma glucose and insulin levels during the OGTT and related factors before and after treatment with placebo or AH.

		Placebo (*n* = 30)				AH (*n* = 28)				*p*-Value ^(2)^
	Time	Baseline	12 Weeks	Change Value	*p*-Value ^(1)^	Baseline	12 Weeks	Change Value	*p*-Value ^(1)^	
FPG (mg/dL)	0 min	108.87 ± 19.46	105.53 ± 19.18	−3.33 ± 13.51	0.187	99.25 ± 11.85	99.71 ± 15.98	0.46 ± 8.32	0.770	0.201
PPG (mg/dL)	30 min	179.03 ± 31.58	176.60 ± 28.12	−2.43 ± 24.83	0.596	176.79 ± 28.64	171.04 ± 36.45	−5.75 ± 29.82	0.317	0.646
	60 min	192.63 ± 45.95	201.03 ± 42.06	8.40 ± 23.91	0.064	194.04 ± 50.50	187.89 ± 50.50	−6.14 ± 33.60	0.342	0.061
	90 min	178.43 ± 50.49	184.10 ± 50.51	5.67 ± 21.09	0.152	183.29 ± 50.10	181.71 ± 57.71	−1.57 ± 31.04	0.791	0.308
	120 min	162.23 ± 46.30	157.73 ± 48.43	−4.50 ± 26.05	0.352	162.04 ± 38.93	165.18 ± 48.88	3.14 ± 28.71	0.567	0.292
FPI (μU/mL)	0 min	11.02 ± 10.25	9.71 ± 4.38	−1.31 ± 10.43	0.498	11.54 ± 7.68	11.46 ± 10.29	−0.08 ± 8.91	0.961	0.634
PPI (μU/mL)	30 min	64.44 ± 42.20	54.30 ± 30.00	−10.15 ± 37.46	0.149	74.73 ± 48.26	58.88 ± 39.65	−15.85 ± 24.46	0.002	0.493
	60 min	77.26 ± 48.71	81.99 ± 35.57	4.73 ± 38.72	0.509	88.17 ± 58.47	77.49 ± 52.70	−10.68 ± 38.71	0.156	0.136
	90 min	76.38 ± 46.71	90.44 ± 51.81	14.06 ± 42.61	0.081	97.06 ± 61.78	80.37 ± 52.95	−16.69 ± 52.42	0.104	0.017 *
	120 min	75.00 ± 40.57	73.52 ± 50.60	−1.47 ± 30.89	0.796	90.33 ± 52.31	81.06 ± 59.53	−9.26 ± 67.63	0.475	0.580
Glucose AUC (mg*min/dL)	0–30 min	1052.50 ± 370.45	1066.00 ± 365.50	13.50 ± 336.85	0.828	1163.04 ± 332.53	1069.82 ± 398.69	−93.21 ± 395.48	0.223	0.272
	0–60 min	3377.00 ± 1214.66	3564.50 ± 1188.71	187.50 ± 788.60	0.203	3747.86 ± 1212.95	3462.32 ± 1232.07	−285.54 ± 1122.76	0.190	0.067
	0–90 min	5664.21 ± 2436.36	6175.98 ± 2429.92	511.77 ± 1309.93	0.041	6430.18 ± 2387.04	6017.66 ± 2357.01	−412.52 ± 1636.33	0.193	0.021 *
	0–120 min	7527.82 ± 3627.51	8141.49 ± 3762.74	613.67 ± 1975.17	0.100	8632.50 ± 3328.94	8236.59 ± 3416.24	−395.91 ± 1944.70	0.291	0.055
Insulin AUC (μU*min/mL)	0–30 min	801.35 ± 593.31	668.75 ± 439.79	−132.60 ± 508.26	0.164	947.84 ± 659.49	711.27 ± 545.49	−236.57 ± 339.60	0.001	0.361
	0–60 min	2596.25 ± 1484.61	2421.65 ± 1173.01	−174.60 ± 1137.57	0.407	3045.11 ± 1948.64	2413.02 ± 1697.61	−632.09 ± 953.97	0.002	0.104
	0–90 min	4570.23 ± 2361.96	4716.65 ± 2052.23	146.42 ± 1761.40	0.652	5477.25 ± 3396.11	4498.48 ± 3042.77	−978.77 ± 1858.27	0.010	0.021 *
	0–120 min	6510.34 ± 3145.84	6884.65 ± 3045.46	374.31 ± 2375.69	0.395	7941.70 ± 4582.33	6515.28 ± 4023.42	−1426.41 ± 3049.73	0.020	0.015 *
Glucose	Cmax (mg/dL)	204.73 ± 42.37	210.30 ± 41.81	5.57 ± 20.25	0.143	205.21 ± 43.24	200.75 ± 51.16	−4.46 ± 28.89	0.421	0.129
	Tmax (min)	63.00 ± 27.69	61.00 ± 24.26	−2.00 ± 27.22	0.690	70.71 ± 30.78	65.36 ± 27.15	−5.36 ± 32.71	0.394	0.672
Insulin	Cmax (μU/mL)	105.13 ± 52.92	110.67 ± 55.97	5.55 ± 53.34	0.573	116.68 ± 60.81	102.48 ± 58.33	−14.21 ± 33.35	0.033	0.095
	Tmax (min)	83.00 ± 32.18	85.00 ± 25.02	2.00 ± 19.19	0.573	87.86 ± 28.20	85.71 ± 32.37	−2.14 ± 31.55	0.722	0.552
HOMA-IR		3.09 ± 3.33	2.58 ± 1.39	−0.50 ± 3.41	0.426	2.89 ± 2.01	3.00 ± 3.23	0.11 ± 2.64	0.826	0.449
HOMA-β		86.87 ± 56.07	87.47 ± 39.32	0.60 ± 55.66	0.953	122.36 ± 109.38	117.00 ± 85.59	−5.36 ± 78.40	0.721	0.739
QUICKI		0.34 ± 0.03	0.34 ± 0.03	0.00 ± 0.03	0.662	0.34 ± 0.03	0.34 ± 0.04	0.00 ± 0.03	0.489	0.839
AIR		0.85 ± 0.70	0.71 ± 0.50	−0.14 ± 0.52	0.150	0.82 ± 0.49	0.70 ± 0.44	−0.11 ± 0.34	0.083	0.823
HbA1c (%)		5.80 ± 0.32	5.82 ± 0.33	0.02 ± 0.18	0.616	5.78 ± 0.35	5.90 ± 0.45	0.11 ± 0.24	0.017	0.083
C-peptide (ng/mL)		2.21 ± 1.02	2.06 ± 0.76	−0.15 ± 0.99	0.414	2.26 ± 0.88	2.15 ± 1.07	−0.11 ± 0.77	0.471	0.856
Adiponectin (ng/mL)		6869.62 ± 3340.48	7005.53 ± 3245.06	135.92 ± 1362.84	0.589	7107.41 ± 4998.33	7104.39 ± 4674.17	−3.02 ± 871.81	0.986	0.644

Values are presented as the mean ± SD. ^(1)^ Analyzed by paired *t*-test between baseline and 12 weeks within each group. ^(2)^ Analyzed by independent *t*-test for change value between the groups. FPG, fasting plasma glucose; PPG, postprandial plasma glucose; FPI, fasting plasma insulin; PPI, postprandial plasma insulin; AUC, area under the curve; HOMA-IR, homeostatic model assessment for insulin resistance; HOMA-β, homeostatic model assessment of β cell function; QUICKI, quantitative insulin sensitivity check index; AIR, acute insulin response. * AH group is significantly different from the placebo group at *p* < 0.05 by *t*-test.

**Table 4:** Hematological and blood biochemical parameters before and after treatment with placebo or AH.

	Placebo (*n* = 30)				AH (*n* = 28)				*p*-Value ^(2)^
	Baseline	12 Weeks	Change Value	*p*-Value ^(1)^	Baseline	12 Weeks	Change Value	*p*-Value ^(1)^	
Hematological analysis									
WBC (×10^3^/μL)	5.82 ± 1.17	5.46 ± 1.03	−0.35 ± 0.98	0.057	5.81 ± 1.51	5.25 ± 1.41	−0.57 ± 0.84	0.001	0.378
RBC (×100^3^/μL)	4.66 ± 0.37	4.58 ± 0.39	−0.08 ± 0.15	0.007	4.69 ± 0.38	4.57 ± 0.41	−0.12 ± 0.22	0.008	0.412
Hemoglobin (g/dL)	14.58 ± 1.47	14.35 ± 1.45	−0.23 ± 0.49	0.015	14.19 ± 1.52	13.81 ± 1.75	−0.37 ± 0.61	0.003	0.332
Hematocrit (%)	43.34 ± 3.76	42.39 ± 3.82	−0.95 ± 1.39	0.001	42.48 ± 3.59	41.33 ± 4.27	−1.15 ± 1.93	0.004	0.656
Platelets count (×10^3^/μL)	241.77 ± 46.66	241.90 ± 46.81	0.13 ± 26.09	0.978	253.40 ± 74.64	252.33 ± 81.86	−1.08 ± 30.53	0.854	0.872
Blood biochemical analysis									
ALP (IU/L)	206.07 ± 55.03	201.70 ± 52.33	−4.37 ± 24.08	0.329	190.21 ± 44.41	184.46 ± 43.60	−5.75 ± 21.98	0.178	0.821
AST (IU/L)	25.43 ± 7.50	25.17 ± 4.82	−0.27 ± 6.25	0.817	28.32 ± 12.27	27.57 ± 8.46	−0.75 ± 5.93	0.509	0.764
ALT (IU/L)	27.80 ± 17.16	25.30 ± 12.94	−2.50 ± 8.41	0.114	34.68 ± 22.52	29.43 ± 14.43	−5.25 ± 11.37	0.021	0.297
Total bilirubin (mg/dL)	0.82 ± 0.23	0.78 ± 0.31	−0.04 ± 0.27	0.411	0.81 ± 0.36	0.80 ± 0.36	−0.01 ± 0.21	0.750	0.656
Total protein (g/dL)	7.12 ± 0.41	6.92 ± 0.36	−0.20 ± 0.22	<0.0001	7.23 ± 0.34	6.92 ± 0.29	−0.31 ± 0.29	<0.0001	0.110
Albumin (g/dL)	4.39 ± 0.18	4.29 ± 0.21	−0.11 ± 0.14	0.0002	4.35 ± 0.21	4.20 ± 0.22	−0.15 ± 0.19	0.0002	0.281
gamma-GT (IU/L)	41.90 ± 30.92	39.60 ± 29.39	−2.30 ± 8.07	0.129	33.14 ± 18.22	30.50 ± 14.79	−2.64 ± 10.71	0.203	0.891
BUN (mg/dL)	13.48 ± 2.99	14.69 ± 3.39	1.22 ± 3.55	0.070	13.74 ± 4.62	12.57 ± 2.93	−1.17 ± 3.31	0.072	0.011 *
Creatinine (mg/dL)	0.94 ± 0.18	0.89 ± 0.19	−0.06 ± 0.13	0.019	0.94 ± 0.18	0.85 ± 0.17	−0.09 ± 0.10	<0.0001	0.234
LD (IU/L)	175.60 ± 21.92	173.43 ± 20.74	−2.17 ± 11.74	0.321	174.96 ± 27.74	174.11 ± 23.05	−0.86 ± 15.17	0.767	0.713
CK (IU/L)	113.80 ± 62.43	116.60 ± 69.82	2.80 ± 64.36	0.813	109.75 ± 78.37	113.64 ± 73.96	3.89 ± 38.76	0.600	0.937
hs-CRP (mg/L)	0.93 ± 1.90	1.00 ± 0.98	0.07 ± 1.85	0.846	1.30 ± 1.60	1.07 ± 1.17	−0.23 ± 1.86	0.524	0.550
Urine analysis									
Specific gravity	1.02 ± 0.01	1.02 ± 0.01	0.00 ± 0.01	0.293	1.02 ± 0.01	1.02 ± 0.00	0.00 ± 0.01	0.231	0.796
pH	6.02 ± 1.19	5.83 ± 0.89	−0.18 ± 0.98	0.313	5.70 ± 0.86	5.45 ± 0.52	−0.25 ± 0.87	0.138	0.785

Values are presented as the mean ± SD. ^(1)^ Analyzed by paired *t*-test between baseline and 12 weeks within each group. ^(2)^ Analyzed by independent *t*-test for change value between the groups. WBC, white blood cells; RBC, red blood cells; ALP, alkaline phosphatase; AST, aspartate aminotransferase; ALT, alanine. aminotransferase; gamma-GT, glutamyl transferase; BUN, blood urea nitrogen; LD, lactate dehydrogenase; CK, creatine kinase; hs-CRP, high sensitivity C-reactive protein. * AH group is significantly different from the placebo group at *p* < 0.05 by *t*-test.

**Table 5:** Biochemical parameters of experimental mice.

	NC	Diabetic Mice	
		DC	AHW
Glucose (mg/dL)	112.5 ± 5.2 ^c^	619.6 ± 14.2 ^a^	484.3 ± 41.2 ^b^
Triglyceride (mg/dL)	141.5 ± 5.7 ^c^	202.1 ± 6.2 ^a^	173.4 ± 1.6 ^b^
Total-cholesterol (mg/dL)	200.3 ± 10.6 ^c^	1100.3 ± 25.2 ^a^	926.6 ± 30.7 ^b^
HDL-cholesterol (mg/dL)	103.8 ± 9.5 ^b^	130.3 ± 3.0 ^a^	130.71 ± 5.2 ^a^
LDL-cholesterol (mg/dL)	59.2 ± 4.2 ^c^	929.6 ± 3.0 ^a^	761.2 ± 5.2 ^b^
ALT (IU/L)	2.4 ± 0.6 ^b^	18.7 ± 1.5 ^a^	15.1 ± 0.4 ^a^
AST (IU/L)	25.4 ± 1.5 ^b^	33.4 ± 1.8 ^a^	34.2 ± 3.2 ^a^
Urea-N (mg/dL)	22.2 ± 1.4 ^b^	26.9 ± 0.8 ^a^	26.9 ± 1.8 ^a^
Creatinine (mg/dL)	1.7 ± 0.1 ^b^	2.4 ± 0.0 ^a^	2.3 ± 0.1 ^a^

Data are presented as the mean ± SEM (*n* = 8). ^a–c^ Different letters are significantly different at *p* < 0.05. NC, normal control; DC, diabetic control; AHW, diabetic mice fed water extract of AH.

**Table 6:** Composition of investigational capsules for the two groups.

Component	Placebo (%)	AH (%)
*Allium hookeri* extract (AH)	-	62.5
Microcrystalline cellulose	98.75	36.72
Silicon dioxide	0.50	0.50
Caramel color	0.70	0.26
Red color	0.05	0.02
Total	100	100

## Data Availability

Data is contained within the article and Supplementary Material.

## Supplementary Materials

The following supporting information can be downloaded at: https://www.mdpi.com/article/10.3390/ph16101364/s1, Figure S1. Inhibitory effects of *A. hookeri* on gluconeogenesis and fatty acid synthesis in the liver of C57BL/KsJ-*db/db* mice fed AHR for 8 weeks. Protein expression of gluconeogenesis, (b) Protein expression of fatty acid synthesis GK: glucokinase, GCKR: glucokinase regulatory protein, G6Pase: glucose-6-phosphatase, PCK1 = PEPCK:phosphoenolpyruvate carboxykinase, SREBP-1: sterol response element binding protein, ACC: acyl-CoA carboxylase, FAS: fatty acid synthase. NC: normal control, DC: diabetic control, AHW: water extract of AH.
