# Supplementary material for: Supplementary Effects of Allium hookeri Extract on Glucose Tolerance in Prediabetic Subjects and C57BL/KsJ-db/db Mice
Source: Pharmaceuticals (Basel). 2023 Sep 27;16(10):1364. doi: 10.3390/ph16101364 (PMC10610268; doi:10.3390/ph16101364)
Supplement: Supplementary file 1 [file pharmaceuticals-16-01364-s001.zip › pharmaceuticals-2582021-supplementary.pdf]

pharmaceuticals

MDPI

Supplementary Material: Supplementary effects of *Allium hookeri* extract on glucose tolerance in prediabetic subjects and C57BL/KsJ-db/db mice

Ji-Su Kim, Hyun-Ju Kim, Eun-Byeol Lee, Ji-Hye Choi, Jieun Jung, Hwan-Hee Jang, Shin-Young Park, Ki-Chan Ha, Yu-Kyung Park, Jong-Cheon Joo, and Sung-Hyen Lee

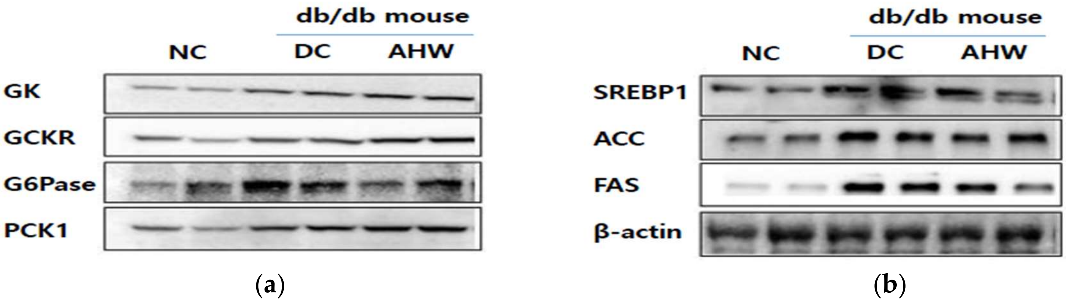

Figure S1. Inhibitory effects of *A. hookeri* on gluconeogenesis and fatty acid synthesis in the liver of C57BL/KsJ-db/db mice fed AHW for 8 weeks. (**a**) Protein expression of gluconeogenesis, (**b**) Protein expression of fatty acid synthesis GK: glucokinase, GCKR: glucokinase regulatory protein, G6Pase: glucose-6-phosphatase, PCK1=PEPCK:phosphoenolpyruvate carboxykinase, SREBP-1: sterol response element binding protein, ACC: acyl-CoA carboxylase, FAS: fatty acid synthase. NC: normal control, DC: diabetic control, AHW: diabetic mice fed hot water extract of AH.
